# Dynamic Gastrointestinal Digestion of Bovine, Caprine and Ovine Milk Reconstituted from Commercial Whole Milk Powders

**DOI:** 10.3390/foods13091403

**Published:** 2024-05-02

**Authors:** Siqi Li, Aiqian Ye, Jian Cui, Yu Zhang, Lara Ware, Jody C. Miller, Holly Abbotts-Holmes, Nicole C. Roy, Harjinder Singh, Warren McNabb

**Affiliations:** 1 Riddet Institute, Massey University, Private Bag 11 222, Palmerston North 4442, New Zealandh.singh@massey.ac.nz (H.S.);; 2 Department of Human Nutrition, University of Otago, Dunedin 9054, New Zealand; lara.ware@otago.ac.nz (L.W.);; 3 High-Value Nutrition National Science Challenge, Liggins Institute, University of Auckland, Auckland 1023, New Zealand

**Keywords:** milk powder, cow milk, goat milk, sheep milk, in vitro digestion, amino acids, digestibility

## Abstract

The global dairy market has been increasingly diversified with more dairy product offerings of milk products from different animal species. Meanwhile, milk powders remain the main exported dairy product format due to their ease of transportation. In this work, we studied the structural changes, protein hydrolysis and nutrient delivery during dynamic gastric digestion and small intestinal digestion of cow, goat and sheep milk reconstituted from commercial whole milk powders. The results show that the reconstituted milks digest similarly to processed fresh milk. The digestion behaviors of the three reconstituted ruminant milks are broadly similar (gastric coagulation, kinetics of gastric emptying of protein and fat and the high digestibility in the small intestine) with some differences, which are likely contributed by the processing history of the milk powders. The delivery of individual amino acids to the small intestine differed between the early and late stages of gastric digestion, which were primarily affected by the abundance of amino acids in caseins and whey proteins but also by the difference between milk types associated with their gastric coagulation behaviors. This work showed that powdered milk is similar to fresh processed milk in digestion behavior, and the inherent differences between ruminant milks can be modified by processing treatments.

## 1. Introduction

Dairy is an important part of the human diet from both cultural and nutritional perspectives. Milk is known to be an important source of high-quality and highly digestible proteins and calcium [1,2,3]. Following consumption, milk components undergo unique physical, chemical and biochemical changes in the gastrointestinal tract that determine nutrient release and absorption. The unique behavior of milk coagulation in the stomach has been studied extensively, which plays a key role in the kinetics of nutrient delivery to the small intestine for absorption [3,4,5,6]. Various factors play a part in the digestion of milk, including milk composition (e.g., presence of fat, casein: whey protein ratio, calcium), processing treatments (e.g., heating, homogenization, high-pressure processing) and dairy animal species [7,8,9,10,11].

Milks from different animals differ considerably in their composition and physicochemical properties [7,12]. Studies on the in vitro digestion of non-bovine milks demonstrated similarities and differences in their gastric coagulation, protein hydrolysis and gastric emptying [8,13,14,15]. Our recent studies investigated the dynamic gastric digestion of differently processed goat and sheep milk in comparison with cow milk [8,16,17]. We found that homogenization and heat treatments accelerated the gastric emptying and digestion of proteins consistently for all species by loosening the gastric coagulum structure. Some differences between species were also found, such as the greater effect of homogenization (two stages, 20 and 5 MPa) in loosening the milk curds formed by goat and sheep milk compared to the curds formed by cow milk [8]. It is of interest to better understand and modulate the digestion behavior and nutrient delivery of milks from different species.

In the global dairy market, a significant portion of milk is processed into milk powders and exported to other countries because of the ease of transporting and storage. Although much research has been conducted in understanding milk digestion, many past studies were conducted on fresh milk. Given the known effects of milk processing on its digestion, there is a lack of understanding of the digestion behavior of powdered milk given the additional steps of processing (evaporation, spray drying), storage and reconstitution before consumption. Past studies have shown that typical powder processing has minimal impact on the digestibility of milk [3,18], but the understanding of whether powdered milk has different structural changes and different nutrient release kinetics from fresh milk is lacking.

This study aims to investigate and compare the gastrointestinal digestion of cow, sheep and goat milk reconstituted from commercial whole milk powders produced in New Zealand. Additionally, we conducted a study using a dynamic gastric digestion model (human gastric simulator) with the same method used to study fresh cow, goat and sheep milk as in previous works [8,16,17] to compare the difference between reconstituted and fresh milks. We hypothesize that reconstituted milks digest similarly to fresh homogenized and pasteurized milk. Finally, we conducted an amino acid analysis of the digesta emptied to the small intestine at the early and late stages of gastric digestion to investigate the role of milk types and gastric emptying kinetics on amino acid delivery.

## 2. Materials and Methods

### 2.1. Milk Powders and Other Materials

Whole milk powders made from cow, goat and sheep milk were used in the study. Cow milk powder was provided by Miraka Ltd. (Taupo, New Zealand). Goat milk powder was provided by NIG Nutritionals Ltd. (Auckland, New Zealand). Sheep milk powder was provided by Spring Sheep Milk Co. (Hamilton, New Zealand). For all the milk powders, the milk was pasteurized and homogenized during the manufacturing process and then spray-dried. The compositions of the milk powders were determined at the Massey University Nutrition Laboratory (Palmerston North, New Zealand) and presented in Table 1. The cow milk powder was higher in carbohydrate and lower in fat and crude protein than the goat and sheep milk powder. In contrast, the sheep milk powder was lowest in carbohydrate and highest in protein and fat. The goat milk powder was intermediate in fat content and closer to the sheep milk powder in protein and carbohydrate contents.

Porcine pepsin (P7000), pancreatin from porcine pancreas (8 × USP specifications, P7545) and bile bovine (B3883) used in digestion studies were purchased from Sigma-Aldrich, St. Louis, MO, USA. All other chemicals were of analytical grade and were purchased from BDH Chemicals (BDH Ltd., Poole, UK) or Sigma Chemical Co. (St. Louis, MO, USA) unless otherwise specified.

### 2.2. Preparation of Reconstituted Milk

To prepare for the digestion experiment, the milk powders were reconstituted to the total solid contents of the raw milk of the different species [7,8]. Reconstituted cow milk (RCM) and reconstituted goat milk (RGM) were reconstituted to 14.0% (*w*/*w*), and reconstituted sheep milk (RSM) was reconstituted to 17.5% (*w*/*w*) in water. The milk was reconstituted in warm water and mixed for 30 min with a magnetic stirrer. Liquid milk samples were preserved with sodium azide (0.02% *w*/*w*). The milk was then stored overnight at 4 °C and used for digestion experiment the next day.

### 2.3. Dynamic In Vitro Gastric Digestion

Gastric digestion experiment was conducted on a human gastric simulator that simulates gastric motility, gradual addition of gastric fluid and gastric emptying. A method described in our previous work [8] used for studying fresh cow, goat and sheep milks was used in this study on reconstituted milks of the three species. Simulated salivary fluid (SSF) and simulated gastric fluid (SGF) were prepared as described according to the INFOGEST recommendations [19] with modifications. No amylase was added to the SSF, and no gastric lipase was added to the SGF. The SGF was prepared to 1.25 × electrolyte concentration and adjusted to pH 1.5. During the dynamic digestion, the electrolyte SGF was added gradually with additional water, CaCl2 and pepsin (P7000, Sigma-Aldrich, St. Louis, MO, USA) to reach the final 1 × concentration and a pepsin activity of 2000 U/mL.

Gastric digestion was conducted for 240 min at 37 °C; 200 g of milk was used in each gastric digestion experiment. The milk was pre-warmed to 37 °C before the experiment. Oral digestion was simulated by mixing SSF with each milk in a 1:1 ratio to the solids content of the milk. For 200 g of RCM, RGM and RSM, the amounts of SSF added were 28, 28 and 35 g, respectively. At the beginning of the gastric digestion, 20 mL of SGF was added to the milk–SSF mixture in the gastric chamber as the basal amount of SGF in the fasted state. For the digestion experiment of RCM, RGM and RSM, the addition rates of SGF were 2.5, 2.5 and 3.0 mL/min and the gastric emptying rates were 3.0, 3.0 and 3.6 mL/min (empties every 20 min), respectively. The emptied digesta was passed through a 1 mm sieve to simulate gastric sieving [20]. These digestion conditions, set differently for the different milk types, were previously tested to allow reaching a final pH of approximately 2.0 by the end of the 240 min digestion period.

The coagulum retained in the stomach collected at 20, 120 and 240 min of digestion were analyzed for microstructure, weight, moisture content and rheological properties. Confocal laser scanning microscopy (CLSM) was performed as described previously [21] after staining the sample with Fast Green and Nile Red. The moisture content was analyzed by oven drying at 105 °C for 24 h and was duplicated for each sample. The rheological analysis was conducted as described by Li et al. [8] with a rheometer (AR-G2; TA Instruments, Crawley, West Sussex, UK) paired with a parallel plate geometry (40 mm diameter).

The liquid-emptied digesta were collected every 20 min after passing through a 1 mm sieve. Each emptied digesta sample was measured for pH, and selected samples (20, 60, 120, 180 and 240 min) were analyzed further for microscopy imaging with CLSM as described above. They were also analyzed for particle size distribution using a MasterSizer 2000 (Malvern Instruments Ltd., Malvern, UK) and for their protein and fat contents with Dumas and Mojonnier method, respectively, as described previously [16]. The emptying and proteolysis during gastric digestion were investigated using glycine sodium dodecyl sulfate–polyacrylamide gel electrophoresis (SDS-PAGE) as described earlier [21]. Milk and digesta samples were diluted in the sample buffer to the same protein concentration. The final gels were scanned using a Gel Doc XR+ (Bio-Rad Laboratories, Hercules, CA, USA).

### 2.4. Delivery of Amino Acids to the Small Intestine and In Vitro Small Intestinal Digestion

Following gastric digestion, the delivery of amino acids to the small intestine and intestine digestion experiment was conducted on pooled digesta emptied in the early or later stages of gastric digestion. From the digesta collected every 20 min during gastric digestion, equal aliquots of every digesta emptied from 20 to 120 min were pooled as the early-emptying gastric digesta (labeled as GE), whereas those emptied from 140 to 240 min were pooled in equal proportions as the late-emptying sample (labeled as GL).

The pooled digesta samples GE and GL were analyzed by SDS-PAGE, and the concentrations of protein and fat as described above. They were also analyzed for the amino acid profile using HPLC following acid hydrolysis (AOAC 994.12) [22] at the accredited Massey University Nutrition Laboratory (Palmerston North, New Zealand). Tryptophan and cystine were not included in the amino acid profile. In addition to the samples, pepsin used in the digestion experiment was also analyzed for amino acid profile as the other source of amino acids in the digesta samples to allow a better interpretation of the results and the difference in amino acid delivery between different milk types and different stages of digestion.

The pooled samples GE and GL were used for intestinal digestion for 120 min using a pH-stat titrator (Titroline 7000, SI Analytics, Mainz, Germany) as described by Pan et al. based on the INFOGEST protocol [19,23]. The pH was maintained at 7.0 by titrating with 0.05 M NaOH. To better estimate free fatty acid release during intestinal digestion, the contribution of proteins and simulated intestinal fluid components to the amount of NaOH titrated was tested using control samples of reconstituted skim cow milk of 0 to 4% protein. A linear standard curve was then established and used to subtract titration volume based on the crude protein content of each digesta sample. The corrected titration volume was used to calculate the amount of free fatty acid (FFA) released during intestinal digestion. In addition, the intestinal digestion samples were also analyzed using SDS-PAGE.

### 2.5. Statistical Analysis

The digestion experiments were triplicated from milk reconstitution to digesta analysis. Minitab 19 was used for statistical analyses. Error bars in the figures indicate standard deviations. One-way analysis of variance (ANOVA) and Tukey’s post-hoc test were used to determine significant differences between groups.

## 3. Results

### 3.1. Gastric Coagulation

**Figure 1:**
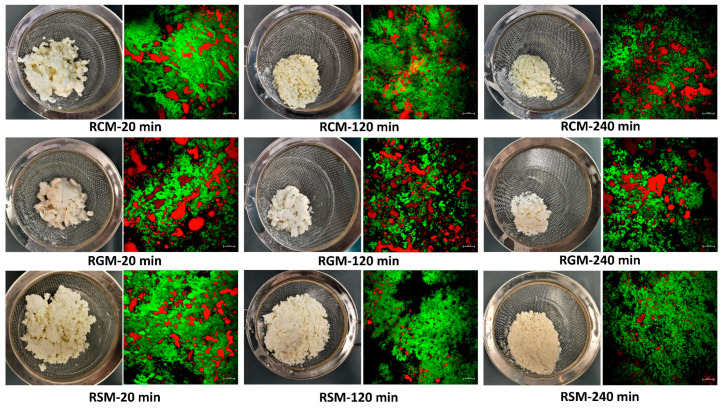
Photos and confocal laser scanning microscopy images of the curds retained in the stomach chamber at different time points during dynamic gastric digestion. In the microscopy images, green indicates protein and red indicates fat; scale bars at the bottom right corner indicate 20 µm. RCM, reconstituted cow milk; RGM, reconstituted goat milk; RSM, reconstituted sheep milk.

Figure 1 shows the photographs and CLSM images of the milk curds retained in the stomach chamber at 20, 120 and 240 min of gastric digestion. All three types of milk formed loose and crumbly curd particles that were larger during early digestion but became finer during later stages of digestion. The microstructures of the curds were also broadly similar between milk types and consisted of a protein matrix with fat embedded in it. During prolonged digestion, the protein networks appeared to become looser and less tight compared with those at 20 min digestion. Some minor differences were found between the three types of milk. At the macro scale, RGM formed larger and more intact curd structures than RCM and RSM at 20 and 120 min. Fat coalescence was most pronounced in the RGM curds, particularly at 120 and 240 min of digestion.

**Figure 2:**
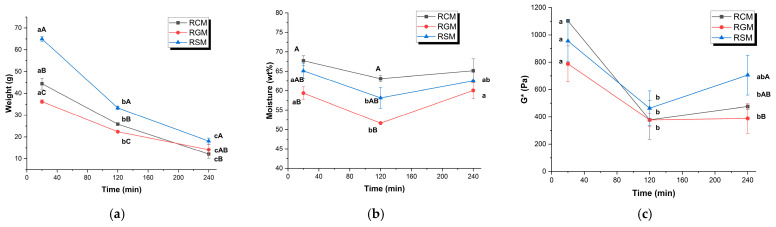
Properties of curd retained in the stomach at 20, 120 and 240 min of digestion: (**a**) wet weight (g); (**b**) moisture content (%); (**c**) consistency indicated by complex modulus G* (Pa). RCM, reconstituted cow milk; RGM, reconstituted goat milk; RSM, reconstituted sheep milk. ^a–c^ Different lowercase letters indicate significant differences over digestion time of the same milk. ^A–C^ Different uppercase letters indicate significant differences between milk types at the same time point.

The weights of fresh curds are presented in Figure 2a. The RSM expectedly formed a higher amount of curd due to its higher solid concentration. The curd weights of RCM and RGM were largely similar, particularly towards the end of gastric digestion. At 20 and 120 min, the curd weight of RCM was higher than that of RGM (*p* < 0.05). This was partially contributed by the different moisture contents of the curds presented in Figure 2b. Throughout the digestion process, the moisture content of the curds formed by the different milks followed the trend of RCM > RSM > RGM. The difference was more pronounced during the first 120 min of digestion and not significant at 240 min. Over the digestion time, the moisture content of the curds all displayed trends of decreasing in the first 120 min, which was significant for RGM and RSM (*p* < 0.05). From 120 to 240 min, the moisture content of RGM curds increased significantly, which was less pronounced for RCM and RSM.

The complex modulus G* of the curds is presented in Figure 2c. Over digestion time, the G* values underwent similar changes for all three types of milk. The G* of the curds was the highest at 20 min and decreased significantly by 120 min. By 240 min, the G* of RSM was higher than RGM. 

### 3.2. Gastric Digesta Emptied to the Small Intestine

#### 3.2.1. pH and Composition

**Figure 3:**
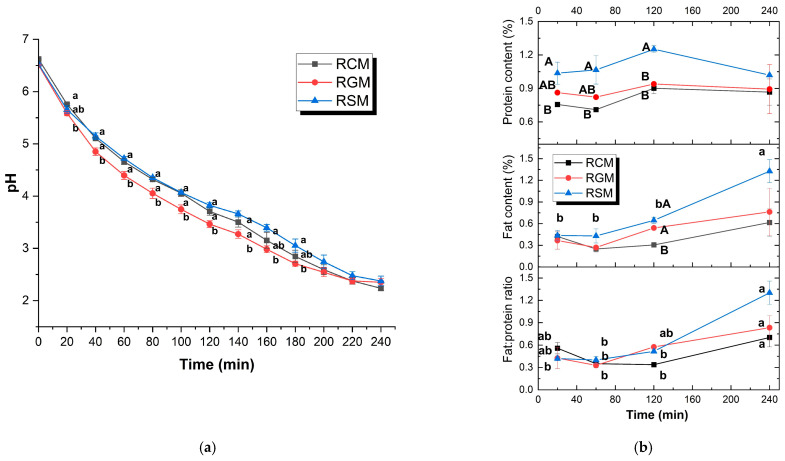
(**a**) pH of gastric digesta sampled at each gastric emptying point; (**b**) protein content, fat content and the fat: protein ratio of digesta emptied at 20, 60, 120 and 240 min of gastric digestion. RCM, reconstituted cow milk; RGM, reconstituted goat milk; RSM, reconstituted sheep milk. ^a,b^ Different lowercase letters indicate significant differences over digestion time of the same milk. ^A,B^ Different uppercase letters indicate significant differences between milk types at the same time point.

The pH profiles were largely similar for the three reconstituted milks during gastric digestion (Figure 3a). The pH decreased rapidly in the first 40 to 60 min, which then slowed down slightly and decreased in a broadly linear fashion till the end of digestion. There were some minor differences between the milk types. Firstly, the pH reduction during the early digestion of RGM was the fastest. From 40 min of gastric digestion, the pH of RGM was significantly lower than those of RCM and RSM, which lasted until 140 min. Secondly, there were slight upward trends in the pH curves at around 140 min for all three milk types, which was most pronounced for RSM.

The composition of the emptied digesta sampled at different time points is presented in Figure 3b. For all three types of milk, the protein contents of the digesta did not change drastically over the course of digestion, with a slightly increasing trend from 60 to 120 min. The fat content of the emptied digesta did not vary significantly over time for RCM or RGM but was significantly higher for RSM at 240 min than earlier time points. The fat: protein ratios of all three types of milk were the highest at 240 min (*p* < 0.05). Between the milk types, RSM had higher protein content than RCM and RGM in the first 120 min (*p* < 0.05). The fat contents were less variable between milk types, except for RCM digesta had lower fat at 120 min than RSM and RGM.

#### 3.2.2. Microstructure and Particle Size Distribution

**Figure 4:**
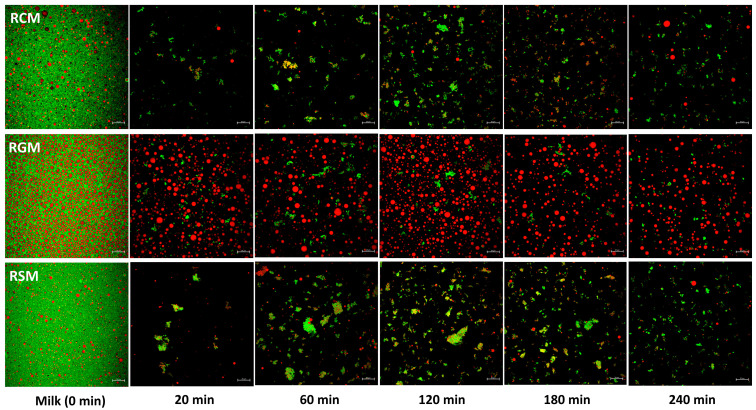
Confocal laser scanning microscopy images of milk and digesta emptied at different time points during dynamic gastric digestion. Green indicates protein; red indicates fat; scale bars at the bottom right corner indicate 20 µm. RCM, reconstituted cow milk; RGM, reconstituted goat milk; RSM, reconstituted sheep milk.

The CLSM images of RCM, RGM, RSM and their gastric digesta emptied at different stages of gastric digestion are shown in Figure 4. Comparing the undigested milks, RGM stands out as it contained more large fat globules (more visible at the scale of the micrograph) than RCM and RSM. This carried over into the digestion process as well that the RGM digesta contained more visible large fat globules than RSM and RCM throughout gastric digestion. In addition to the fat globules, the RGM digesta contained some protein aggregates, particularly at 120 min. The change in particle structures in the emptied digesta of RCM and RSM over time were similar. Protein aggregates increased in size and abundance from 20 to 120 min and slightly decreased in size towards the end of digestion. Only small amounts of visible large fat globules were present. The only apparent difference between RCM and RSM was the overall larger protein particle size of RSM.

**Figure 5:**
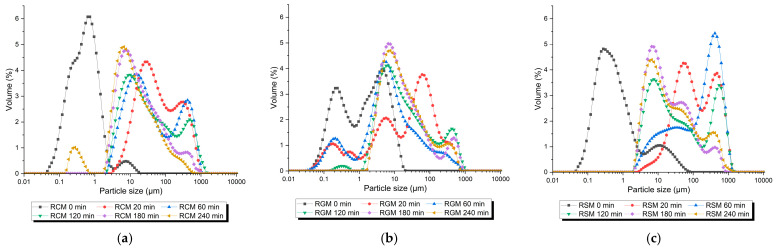
Particle size distribution of milk and gastric digesta emptied at different stages of dynamic gastric digestion: (**a**) reconstituted cow milk (RCM); (**b**) reconstituted goat milk (RGM); (**c**) reconstituted sheep milk (RSM).

The particle size distribution results presented in Figure 5 are consistent with the microscopy images. Particles in undigested reconstituted milks (black squares in Figure 5) consisted of three populations, the casein micelles at around 0.2 µm, insoluble large particles of around 10 µm, and homogenized fat globules in between the two populations, in agreement with those reported previously for reconstituted whole milk powder [24]. Consistent with the microscopy images (Figure 3), the population of fat globules in RGM was the largest (shoulder peak at around 2 µm), merging with the larger particle population at around 10 µm. The fat globules were smaller in RCM and RSM (<1 µm). During gastric digestion of all three milks, the particle size distribution of the emptied digesta shifted to the larger side in the first 60 to 120 min of digestion, reaching the maximum of 1000 µm that matches with the simulated gastric sieving size (1 mm). During later stages of digestion at 120 to 240 min, the large particle population of over 100 µm shrank and shifted to a smaller size. The main particle population towards the end of gastric digestion for all three milks centered at around 10 µm. Despite the similar overall pattern, differences between milk types were pronounced in the evolution of particle size distribution over time. The increase in the large particle population (close to 1000 µm) was fastest for RSM, followed by RCM and was slowest for RGM. The relative volume of large particles also peaked highest for RSM Figure 5c (RSM 60 min), followed by RCM and then RGM.

#### 3.2.3. Protein Hydrolysis in Gastric Digesta

**Figure 6:**
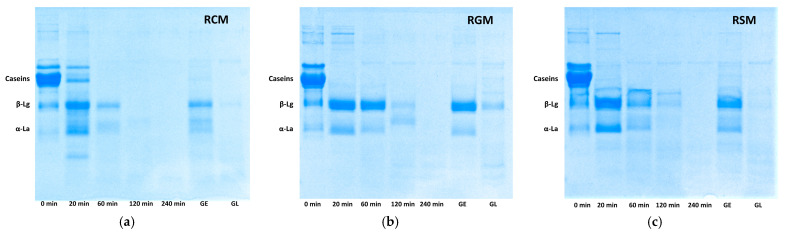
SDS-PAGE profiles of milk and emptied gastric digesta. (**a**) Reconstituted cow milk (RCM). (**b**) Reconstituted goat milk (RGM). (**c**) Reconstituted sheep milk (RSM). GE, early-emptying gastric digesta (20–120 min); GL, gastric late-emptying (140–240 min); M, molecular weight marker.

Figure 6 shows the SDS-PAGE gel patterns of the reconstituted milks and emptied gastric digesta. Overall, digesta of all milk types consisted of mainly intact whey proteins in the first 60 min of digestion in contrast to the original milks that were abundant in caseins, indicating the gastric coagulation of casein micelles and the early emptying of whey proteins. From 120 min, only faint whey protein bands and peptide bands were visible, indicating high levels of proteolysis during the later stages of gastric digestion. The RCM digesta at 20 min contained more intact caseins than RGM and RSM, suggesting more caseins were emptied from the stomach and did not coagulate into curds during early digestion. At 120 min of digestion, only the RGM digesta contained a β-Lg band, which was a faint band in RSM and absent in RCM digesta at 120 min.

The gastric digesta emptied early in the first 120 min of digestion (GE) contained more intact whey proteins, whereas those emptied later in the last 120 min of digestion (GL) contained little intact protein and more small peptides. The GL of RGM contained a clear β-Lg band that was very faint for RCM and RSM. These samples were used for intestinal digestion and amino acid studies, as presented below.

### 3.3. Nutrient Release from the Stomach and Intestinal Digestion

#### 3.3.1. Intestinal Digestion

**Figure 7:**
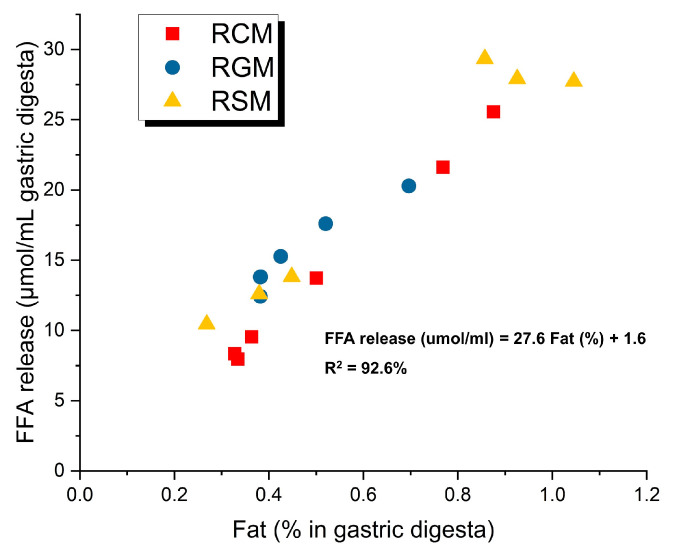
Relationship between estimated free fatty acid release during intestinal digestion and the fat contents of gastric digesta of different reconstituted ruminant milks. Linear regression results of all data points are presented. FFA, free fatty acids; RCM, reconstituted cow milk; RGM, reconstituted goat milk; RSM, reconstituted sheep milk.

The early-emptying (0–120 min) and late-emptying (120–240 min) gastric digesta of the three reconstituted milks were used for simulated intestinal digestion for 120 min. For studying lipid digestion, FFA release during intestinal digestion was calculated based on pH-stat titration volume and subtraction of fat-free standards, and the results are presented in Figure 7. FFA release was expectedly found to correlate with the amount of fat in the pooled gastric digesta (R^2^ = 0.93), indicating that the milk types did not impact milk lipid digestibility. In addition, most of the titration during intestinal digestion took place in the first 10 min of digestion time, indicating a rapid lipid digestion process.

Similarly, milk proteins were also found to be highly and rapidly digested in the small intestine. All of the intestinal digesta showed no visible bands of milk proteins or peptides on SDS-PAGE gels (results not shown). This is expected, as milk proteins are known to be highly digestible [3,25]. For this reason, to evaluate the possible difference in the delivery of amino acids between milk types and stages of digestion, we analyzed the amino acid profiles of gastric digesta used in small intestinal digestion.

#### 3.3.2. Amino Acid Delivery

The amino acid profiles of the three types of reconstituted milks were largely comparable (Table 2), as reported previously [26], with some differences between the species. The amino acid profile of RGM had the most different features compared with RCM and RSM, including the highest threonine and valine and the lowest tyrosine contents, all agreeing with that reported by Landi et al. [27]. Additionally, RGM had a slightly higher proportion of proline than RCM and RSM; RCM contained lower lysine content than RGM and RSM. Meanwhile, higher proportions of aspartic acid and alanine were found in the amino acid profile of RSM.

The amino acid profile of pepsin used in the digestion study is also presented in Table 2 for the interpretation of the dynamic digestion effects on the amino acid profile of the digesta at different stages. During dynamic digestion, the SGF (contains pepsin) was gradually added, whereas the food chyme in the stomach was diluted and emptied over time. Consequently, the amino acid profiles of digesta emptied from the stomach would more resemble that of pepsin over digestion time. By comparing the amino acid profiles of undigested milk, pepsin and the digesta sampled from the early and later stages of digestion (Table 2), we could understand which amino acids were emptied earlier or later during digestion. This works particularly well for those amino acids that are present in markedly different percentages in pepsin than in the milks. Based on the results, the amino acids are categorized into three groups: early emptying, late emptying and the rest that empties consistently during digestion.

The early-emptying amino acids include aspartic acid, threonine, alanine and, to a lesser extent, isoleucine and lysine. For aspartic acid, threonine and alanine, all of them were higher in the early digesta than in the undigested milks or late digesta, despite the higher contents of these amino acids in pepsin that promoted higher concentrations towards later stages of digestion. It was evident that these three amino acids were emptied early during gastric digestion. For isoleucine and lysine, the early digesta tend to be richer in these amino acids than the late digesta, but the pattern was less pronounced and was also confounded by a lower lysine percentage in pepsin than in the milk. Between the milk types, RGM showed a more pronounced and consistent early-emptying effect of these amino acids than RCM and RSM.

The late-emptying amino acids include tyrosine, proline and phenylalanine. These amino acids were generally higher in the late digesta than in the early digesta, despite their lower concentrations in pepsin than in the milks. The late-emptying effect was most pronounced for tyrosine and significant for all three milk types (Table 2). For proline and phenylalanine, the late emptying effect was most pronounced for RGM and least pronounced for RCM.

Other amino acids did not display a clear early- or late-emptying pattern, including serine, glutamic acid, glycine, valine, methionine, leucine, histidine and arginine. The concentrations of these amino acids either did not vary significantly or shifted clearly from that of the original milks to that of pepsin over digestion time. In the latter case, the effect of increasing the proportion of pepsin in the digesta dominated the change in the concentration of these amino acids (e.g., glycine) over time. However, the lack of difference between early and late digesta in methionine and glutamic acid, despite their much lower concentrations in pepsin than in the milks, suggested that they may have been emptied more later during digestion.

Among the milk types, milks rich or poor in certain amino acids also tend to contain a greater amount of these amino acids in the digesta, such as the higher threonine and valine and lower tyrosine in RGM digesta (both early and late stages) than RCM and RSM. Interestingly, RCM, being richest in proline had the lowest mean proline concentration in the early-stage digesta among the three milks and significantly higher proline in late-stage digesta than RCM and RSM.

## 4. Discussion

Previous studies have shown that processing treatment and animal species have significant impacts on the gastric coagulation behavior of the milk, which consequently greatly affects the rate of gastric emptying and digestion of milk proteins and lipids [8,15,16,17,21,24,28]. It is of interest to compare the digestion behaviors of milk of different species (RCM, RGM and RSM) as well as between reconstituted milks from powders in the present study and the corresponding processed fresh milk of the same species studied using the same gastric digestion method [8].

### 4.1. Comparison of Gastric Digestion Behaviors between the Three Types of Reconstituted Milk

Overall, we found a range of similarities between the three types of milk investigated despite some minor between-species differences. The similarities include the following.

1. Decrease in curd moisture in the first 120 min of digestion (Figure 2b), as previously demonstrated. This is due to the structural compaction and moisture repulsion process in the milk coagulum during gastric digestion [29]
2. Similar change of G* of the curds over time (Figure 2c), which could be associated with the dissolution of colloidal calcium phosphate and restructuring of the curd as the digestion progressed and the pH decreased.
3. Similar pH curves and the increase in fat: protein ratios over digestion time, indicating similar coagulation and breakdown patterns of the milk curds during digestion.
4. Similar pattern of increasing particle size in the emptied digesta in the first 120 min of digestion and decrease afterwards. Particle size increases initially because milk proteins coagulate due to the milk clotting activity of pepsin at high pH levels [30], whereas the decrease in particle size is caused by the protein hydrolysis by pepsin as its proteolytic activity increases significantly at about pH 4 [31].

Some differences were also found between the milk types. The RGM curds were more intact and lower in moisture during the early stages of digestion (Figure 1 and Figure 2b), which likely contributed to the slightly faster initial pH drop (Figure 3a) due to the depletion of the major buffering component caseins. This contrasted previous studies showing that goat milk formed a loose and more hydrated gastric coagulum compared with cow milk [8,32]. One likely reason for the stronger initial coagulation of RGM in the present study is its larger fat globule size (Figure 4), probably attributed to the milder homogenization conditions of the commercial goat milk powder. It was demonstrated previously that the homogenization of milk results in loose milk curds during gastric digestion that are more hydrated [8,21,28]. Non-homogenized pasteurized milk forms intact and dense milk clots during gastric digestion [8,14,21], similar to raw milks. The fat globule size of RGM was around 2 µm (Figure 5b), much larger than the homogenized fresh goat milk in earlier studies [8,16]. Besides the probable effect of fat globule size, the higher protein concentration of goat milk powder than cow milk powder may also play a role in the greater coagulation of RGM (Table 1), which is different from the protein concentrations of raw goat and cow milks reported previously in New Zealand [8,14]. This finding indicates that processing has a greater effect on the gastric digestion behavior of ruminant milks than animal species and can be used to effectively control digestion kinetics.

The increase in particle size in the digesta (Figure 4 and Figure 5), presumably caused by the coagulation of proteins as described above at a high pH, was greatest for RSM, followed by RSM, and lowest for RGM. Different factors may contribute to this difference. The RSM was prepared to be higher in solids and proteins, which could help with promoting protein aggregation. For RGM, it is apparent that greater curd formation retained in the stomach, as discussed above, may have depleted the proteins available in the liquid phase that can be emptied.

### 4.2. Comparison of In Vitro Digestion Behaviors between Reconstituted and Fresh Milk

We found all three types of reconstituted milk coagulated during gastric digestion into loose curds similar to homogenized and heated (75 °C for 15 s to 95 °C for 5 min) fresh cow, goat and sheep milk, as reported previously [8]. Moreover, many consistent digestion behaviors of the reconstituted milks in this study discussed above are also consistent with past studies on fresh processed cow, sheep and goat milk using the same digestion protocol, including the decrease in curd moisture, changes in the complex modulus of the curds and the increasing fat to protein ratio over digestion time [8,16,17]. Overall, whole ruminant milks reconstituted from powders displayed largely the same digestion behaviors to commonly processed fresh milk that underwent homogenization and pasteurization. Homogenization and heat treatment during milk powder processing appear to be the main steps determining the digestion behaviors, whereas spray-drying and reconstitution do not have a noticeable impact.

### 4.3. Intestinal Digestion and Amino Acid Delivery

We, for the first time, investigated the difference in amino acid delivery to the small intestine in the dynamic Human Gastric Simulator by comparing the amino acid profiles of the original milks, their digesta emptied in the early or later half of digestion and pepsin. The amino acids that displayed distinct gastric emptying patterns may be explained primarily by their different abundance in caseins and whey proteins [33]. The most pronounced early emptying amino acids aspartic acid, threonine and alanine are much richer in whey proteins than in caseins. Lysine and isoleucine, which also showed early-emptying trends, are also richer in whey proteins than in caseins, but the difference is smaller. In contrast, the late-emptying amino acids tyrosine, proline and phenylalanine are much richer in caseins than in whey proteins. The lack of significant differences in methionine and glutamic acid, which are richer in caseins between early- and late-stages of digestion despite the much lower concentration of these amino acids in pepsin than in milk, also indicate their tendency to be emptied later during digestion.

From a nutritional perspective, caseins have known as “slow” proteins, whereas whey proteins are “fast” proteins due to their different kinetics of digestion and absorption [34]. The gastric coagulation of milk plays a major role in determining the kinetics of amino acid deliver to the small intestine. Caseins coagulate into clots and retain longer in the stomach and those amino acids present in caseins in high proportions were emptied at a later stage. Despite some whey protein denaturation in commercially produced milk powders, which cause their incorporation into the clots and delayed emptying and digestion [3,35], the majority of whey proteins were emptied during early digestion as demonstrated in the SDS-PAGE profiles (Figure 6), leading to the amino acids richer in whey proteins being emptied early during digestion. In addition to the compositional difference between caseins and whey proteins, a minor factor that may have contributed to the emptying kinetics of different amino acids is hydrophobicity. Past studies showed that peptides produced from milk protein digestion associated with the milk clots in the stomach, such as the hydrophobic para-κ-casein [14,16,35]. Following initial digestion of intact proteins, a more hydrophobic amino acid like phenylalanine, may have been more associated with the curds and remain in the stomach for longer compared with a less hydrophobic amino acid like glutamic acid, which contributed to the more pronounced late-emptying behavior of phenylalanine than glutamic acid. However, to better understand this effect, further research is needed to understand the hydrophobicity of peptides released from pepsin hydrolysis of milk proteins, the amino acid composition of these peptides and their tendency to associate with the milk coagulum. For example, the hydrophilic casinomacropeptide contains only one leucine, whereas para-κ-casein, which associates with the gastric protein coagulum, contains the remaining seven leucine in κ-casein [35] The early release of casinomacropeptide and its emptying with whey proteins from the stomach would reduce the concentration of leucine delivered early to the small intestine.

When comparing the milk of difference species, RGM stood out as displaying the most pronounced contrast between the early-emptying and late-emptying amino acids whereas for RSM and particularly RCM, the early- or late-emptying patterns were less significant (Table 2). These difference between milk types may have been associated with their different gastric coagulation behaviors discussed above. The RGM, with its larger fat globule size, had greater incorporation of caseins in the clots during early digestion, and the more intact curd structure delayed the hydrolysis of caseins and the release of casein-rich amino acids. In contrast, the small fat globule size and lower concentration of protein in RCM may have resulted in less coagulation of caseins, as evidenced by the presence of intact caseins in the early RCM digesta. Consequently, the difference between early and late stages of digestion in amino acid composition was least pronounced for RCM.

## 5. Conclusions

In this study, we demonstrated the dynamic gastric digestion behaviors of ruminant milks (cow, goat and sheep) reconstituted from commercial whole milk powders. The results showed that reconstituted whole milks digest similarly to fresh processed consumer milk reported in previous studies. The three types of milk displayed overall similar digestion behaviors and high overall digestibility, with some minor differences in gastric coagulation and gastric emptying of milk solids. Processing treatments of milk during powder manufacture, such as homogenization, appear to have a dominant effect on milk digestion and can be used as an effective leaver to modulate digestion behaviors. The delivery of different amino acids to the small intestine happened at different stages of dynamic gastric digestion, which are primarily affected by the early emptying of whey proteins and late emptying of caseins for all three reconstituted milks with minor differences associated with their amino acid composition and gastric coagulation behaviors.

## Figures and Tables

**Table 1:** Composition of the milk powders (%, *w/w*).

	Cow Milk Powder	Goat Milk Powder	Sheep Milk Powder
Fat	23.6	26.2	29.9
Crude protein	26.7	31.8	33.3
Carbohydrate	41.1	32.7	29.2
Ash	5.4	6.9	5.2
Moisture	3.2	2.5	2.4

**Table 2:** Amino acid profile (% of total quantified amino acids) of the milks of different species, pepsin and gastric digesta emptied during the early and late stages of gastric digestion *.

	RCM	RGM	RSM	RCM-GE	RGM-GE	RSM-GE	RCM-GL	RGM-GL	RSM-GL	Pepsin
Aspartic Acid	7.2 ^d^	7.3 ^d^	7.9 ^cd^	8.9 ^bc^	9.5 ^b^	9.0 ^bc^	8.2 ^cd^	7.7 ^d^	8.2 ^cd^	12.8 ^a^
Threonine	4.1 ^bcd^	4.8 ^ab^	4.1 ^bcd^	4.6 ^bcd^	5.6 ^a^	4.6 ^bc^	3.9 ^d^	4.5 ^bcd^	4.0 ^cd^	5.0 ^ab^
Alanine	3.1 ^e^	3.1 ^de^	3.6 ^cde^	3.8 ^c^	4.6 ^b^	4.7 ^b^	3.9 ^c^	3.6 ^c^	3.9 ^c^	6.2 ^a^
Isoleucine	5.0 ^a^	4.7 ^abc^	4.8 ^ab^	4.9 ^ab^	4.7 ^ab^	4.6 ^bcd^	4.4 ^cd^	4.3 ^d^	4.4 ^cd^	3.6 ^e^
Lysine	7.8 ^cd^	8.5 ^ab^	8.8 ^a^	8.0 ^bcd^	8.3 ^abc^	8.4 ^abc^	7.6 ^de^	7.3 ^e^	8.0 ^bcd^	6.3 ^f^
Tyrosine	5.4 ^a^	4.2 ^b^	5.1 ^a^	4.0 ^bc^	3.5 ^cd^	4.0 ^bc^	5.1 ^a^	4.2 ^b^	5.0 ^a^	3.3 ^d^
Proline	10.6 ^b^	11.4 ^a^	10.6 ^bc^	10.1 ^bcd^	9.6 ^d^	10.0 ^cd^	10.2 ^bc^	11.4 ^a^	10.6 ^b^	8.2 ^e^
Phenylalanine	5.2 ^a^	4.9 ^ab^	4.7 ^ab^	4.3 ^cd^	4.2 ^d^	4.1 ^d^	4.7 ^bc^	4.8 ^ab^	4.5 ^bcd^	3.0 ^e^
Serine	5.4 ^bc^	5.1 ^c^	5.1 ^c^	5.4 ^bc^	5.1 ^c^	5.3 ^bc^	5.8 ^ab^	5.5 ^bc^	5.6 ^bc^	6.4 ^a^
Glutamic Acid	19.5 ^ab^	19.6 ^a^	19.4 ^ab^	19.2 ^bc^	17.8 ^c^	18.5 ^abc^	18.2 ^bc^	18.1 ^c^	18.5 ^abc^	11.2 ^d^
Glycine	1.8 ^d^	1.6 ^d^	1.7 ^d^	4.1 ^c^	4.1 ^c^	3.8 ^c^	4.9 ^b^	4.8 ^b^	4.2 ^bc^	14.1 ^a^
Valine	6.5 ^bc^	7.4 ^a^	6.7 ^b^	6.1 ^de^	6.4 ^c^	6.1 ^d^	5.8 ^e^	6.5 ^bc^	5.9 ^de^	4.3 ^f^
Methionine	2.8 ^a^	2.7 ^ab^	2.9 ^a^	2.2 ^b^	2.2 ^b^	2.3 ^b^	2.4 ^ab^	2.5 ^ab^	2.3 ^b^	1.4 ^c^
Leucine	9.7 ^a^	9.4 ^a^	9.3 ^ab^	8.3 ^c^	8.7 ^bc^	8.5 ^c^	8.3 ^c^	8.4 ^c^	8.4 ^c^	4.7 ^d^
Histidine	2.3	2.1	2.2	2.1	2.2	2.3	2.1	2.4	2.4	2.3
Arginine	3.6 ^def^	3.1 ^f^	3.3 ^ef^	4.0 ^bcd^	3.6 ^de^	3.9 ^cd^	4.3 ^b^	4.2 ^bc^	4.1 ^bcd^	7.2 ^a^

* RCM, reconstituted cow milk; RGM, reconstituted goat milk; RSM, reconstituted sheep milk; GE, pooled gastric digesta emptied during early stages (0–120 min) of digestion; GL, pooled gastric digesta emptied during the late stages (120–240 min) of digestion. ^a–f^ Means within the same row with different superscripts differ significantly (*p* < 0.05, one-way ANOVA with Tukey’s post-hoc test).

## Data Availability

The original contributions presented in the study are included in the article, further inquiries can be directed to the corresponding author.
